# Motor Overflow and Spasticity in Chronic Stroke Share a Common Pathophysiological Process: Analysis of Within-Limb and Between-Limb EMG-EMG Coherence

**DOI:** 10.3389/fneur.2018.00795

**Published:** 2018-10-09

**Authors:** Yen-Ting Chen, Shengai Li, Elaine Magat, Ping Zhou, Sheng Li

**Affiliations:** ^1^Department of Physical Medicine and Rehabilitation, McGovern Medical School, University of Texas Health Science Center – Houston, Houston, TX, United States; ^2^TIRR Research Center, TIRR Memorial Hermann Hospital, Houston, TX, United States

**Keywords:** motor overflow, stroke, spasticity, EMG-EMG coherence, reticulospinal tract

## Abstract

The phenomenon of exaggerated motor overflow is well documented in stroke survivors with spasticity. However, the mechanism underlying the abnormal motor overflow remains unclear. In this study, we aimed to investigate the possible mechanisms behind abnormal motor overflow and its possible relations with post-stroke spasticity. 11 stroke patients (63.6 ± 6.4 yrs; 4 women) and 11 healthy subjects (31.18 ± 6.18 yrs; 2 women) were recruited. All of them were asked to perform unilateral isometric elbow flexion at submaximal levels (10, 30, and 60% of maximum voluntary contraction). Electromyogram (EMG) was measured from the contracting biceps (iBiceps) muscle and resting contralateral biceps (cBiceps), ipsilateral flexor digitorum superficialis (iFDS), and contralateral FDS (cFDS) muscles. Motor overflow was quantified as the normalized EMG of the resting muscles. The severity of motor impairment was quantified through reflex torque (spasticity) and weakness. EMG-EMG coherence was calculated between the contracting muscle and each of the resting muscles. During elbow flexion on the impaired side, stroke subjects exhibited significant higher motor overflow to the iFDS muscle compared with healthy subjects (ipsilateral or intralimb motor overflow). Stroke subjects exhibited significantly higher motor overflow to the contralateral spastic muscles (cBiceps and cFDS) during elbow flexion on the non-impaired side (contralateral or interlimb motor overflow), compared with healthy subjects. Moreover, there was significantly high EMG-EMG coherence in the alpha band (6–12 Hz) between the contracting muscle and all other resting muscles during elbow flexion on the non-impaired side. Our results of diffuse ipsilateral and contralateral motor overflow with EMG-EMG coherence in the alpha band suggest subcortical origins of motor overflow. Furthermore, correlation between contralateral motor overflow to contralateral spastic elbow and finger flexors and their spasticity was consistently at moderate to high levels. A high correlation suggests that diffuse motor overflow to the impaired side and spasticity likely share a common pathophysiological process. Possible mechanisms are discussed.

## Introduction

**Figure 1:**
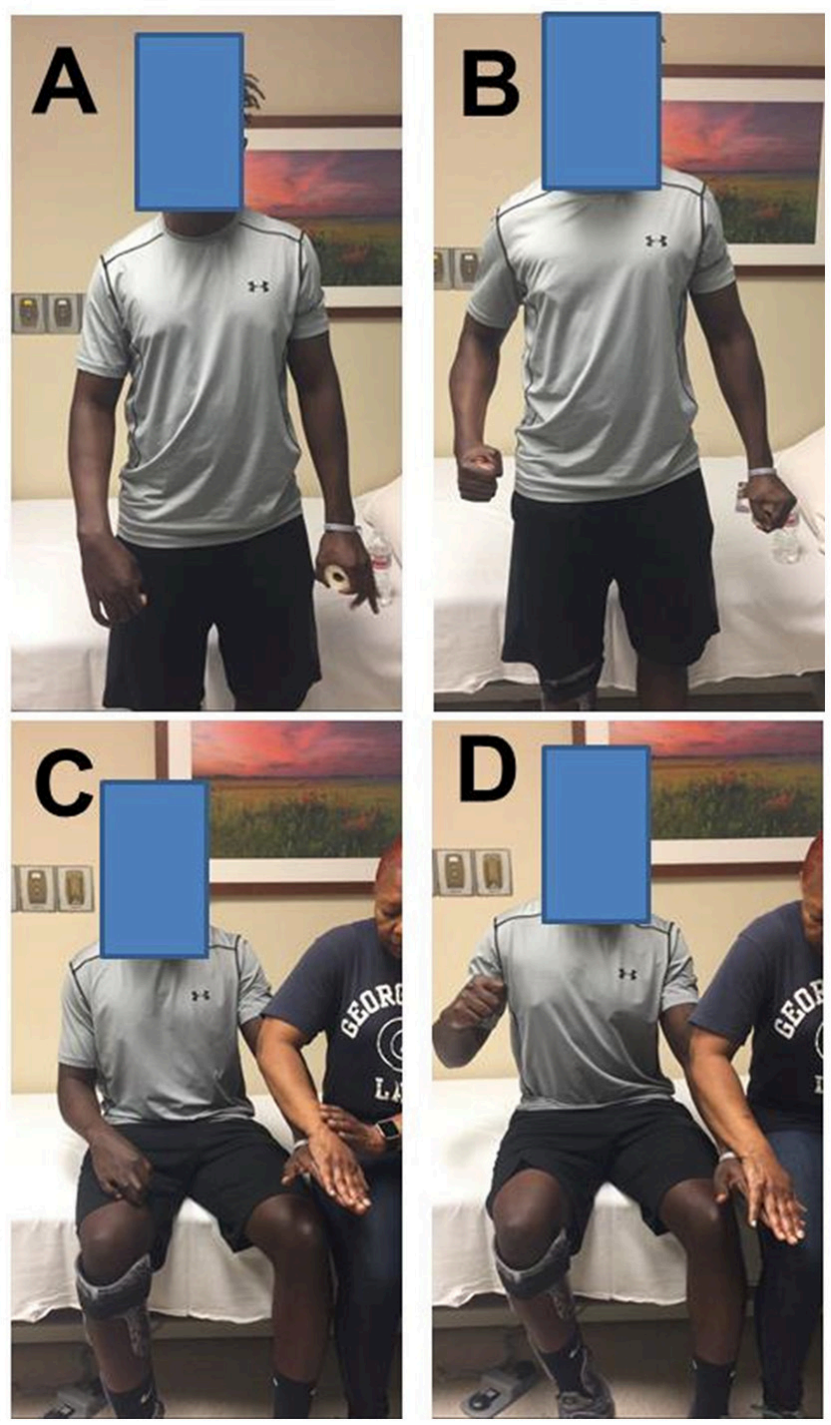
Motor overflow in a 41 year old stroke survivor with right spastic hemiplegia from a left middle cerebral artery hemorrhagic stroke. **(A)** standing and relaxed; **(B)** standing and left hand squeezing; **(C)** sitting and relaxed; **(D)** sitting and resisted hand/finger extension on the left side. Photos were recently taken from PI's spasticity clinic, a written consent of media release was signed by the patient.

When a stroke survivor with spastic hemiplegia is asked to squeeze the hand or flex the elbow joint on the non-impaired side as shown in Figure 1, there is involuntary activation of spastic finger and elbow flexors on the impaired side (Figures 1A, B). This phenomenon of involuntary activation of spastic muscles can occur in about 30% of hemiplegic stroke (1). It is often referred as motor overflow or associated reaction (1–8). Other terms, such as mirror movement, global synkinesis, are sometimes used interchangeably for the same clinical observation (8). Motor overflow is one form of the spastic muscle overactivity. Other types of muscle overactivity are also seen clinically, such as spastic dystonia, co-contraction (9, 10).

Motor overflow is commonly observed in the contralateral homologous resting muscle(s). It can also be seen from proximal muscles to distal muscles in a form of abnormal synergy (11, 12), and between limbs on the impaired side through interlimb coupling (13). As demonstrated in Figures 1C,D, motor overflow to the contralateral spastic finger and elbow flexors occurs during voluntary finger extension on the non-impaired side. These clinical presentations indicate that motor overflow to the spastic muscles is non-selective, diffuse, and concomitantly with voluntary activation of other muscles. In contrast, motor overflow seen in neurologically intact adults is mainly in contralateral homologous muscles in the context of extreme effort or fatigue [see review (14)]. Therefore, motor overflow in stroke survivors is likely mediated by different mechanisms than in healthy adults. However, the underlying mechanisms for motor overflow after stroke are poorly understood.

A number of methods have been used in the literature to evaluate motor overflow after neurological impairments, including surface EMG, goniometry, dynamometry, electrogoniometry, and clinician rating form. Surface EMG is the most commonly used laboratory-based method (8). In our recent studies (15, 16), involuntary EMG activities of the contralateral resting muscles were used to quantify the extent of motor overflow during unilateral voluntary elbow flexion tasks. Using quantitative assessment, the level of motor overflow is found to be graded by the effort of the non-impaired muscles (3). Furthermore, EMG-EMG coherence analysis between EMG signals from the contracting muscle and the contralateral resting muscles could provide potential sources of motor overflow. Coherence analysis is based on the cross-correlation between two separate signals in the frequency domain. Coherence values fall between 0 and 1. Commonly studied frequency bands include 6–12 Hz (alpha band), 13–30 Hz (beta band), and 30–60 Hz (gamma band). It is well accepted that both beta and gamma bands have cortical origins (17–20). Coherence in the alpha band is believed to have subcortical influences, may be related to the reticulospinal drive (21). For example, EMG signals were recorded from bilateral homologous muscles, such as biceps muscles during motoric responses of acoustic startle reflex and during similar voluntary movements in healthy subjects. EMG-EMG coherence in the alpha band was significantly greater during startle reflex responses than during voluntary movement, suggestive of a reticulospinal origin of such coherence in the alpha band (21).

Motor overflow is often seen and elicited in stroke survivors with spasticity. Its relation with post-stroke spasticity remains controversial. Motor overflow is found to be associated with spasticity in some studies (2, 3, 6), but not in others (1, 4). In all these studies, spasticity was assessed using clinical scales, such as modified Ashworth scale or Tardieu scale. Quantitative assessment is likely to provide better insights into this relationship. Based on the velocity-dependent increase in resistance feature of spasticity, a quantitative assessment with computerized control of external stretch was developed (22, 23). In this approach, a joint is stretched by a motorized device at a controlled, constant speed. Resistance torque is obtained to quantify responses from spastic muscles. Reflex torque is quantified objectively by subtracting passive resistance at a very slow speed of stretch, e.g., 5°/s from that at a fast speed, e.g., 100°/s. Reflex torque is attributed primarily to underlying neural mechanisms of spasticity. In a previous study (24), we have demonstrated that reflex torque was velocity-dependent at the same wrist position (muscle length), and changed with various wrist positions at the same speed of stretch. This biomechanical quantification of spasticity is also sensitive to quantify reflex and non-reflex responses from spastic elbow flexors in response to controlled cold exposure (25).

In the present study, the specific aim was to examine the possible mechanisms mediating the phenomenon of motor overflow in chronic stroke. Stroke survivors and healthy controls were instructed to flex the elbow joint voluntarily at submaximal levels. Surface EMG signals were recorded from bilateral elbow flexors and finger flexors to quantify motor overflow. Within-limb and between-limb EMG-EMG coherence analyses were performed. Elbow flexor spasticity was quantified using our established biomechanical approach. Since motor overflow is commonly seen in stroke survivors with spasticity, they may share the same underlying pathophysiology. We hypothesized that there is greater motor overflow to the spastic elbow and finger flexors and that greater motor overflow is highly correlated with spasticity, as compared to the control group. Furthermore, post-stroke spasticity is primarily attributed to reticulospinal hyperexcitability and has separate underlying mechanisms for weakness (26, 27). between-limb intermuscular EMG signals were hypothesized to have significant EMG-EMG coherence in the alpha band to reflect reticulospinal hyperexcitability. Motor overflow was further hypothesized to correlate with spasticity (reflex torque), but not weakness.

## Methods

### Participants

Eleven healthy adults (Age: 31.18 ± 6.18 yrs; 2 women) and 11 stroke patients (63.6 ± 6.4 yrs; 4 women) participated in this study. All healthy subjects reported no known neuromusculoskeletal impairments and were right-handed. Inclusion criteria for the stroke subjects were: (1) hemiplegia secondary to an ischemic or hemorrhage stroke; (2) at least 6 months post-stroke; (3) residual voluntary elbow flexion force; (4) spastic hypertonia in elbow flexors of the impaired side, rated as Modified Ashworth Scale (MAS) less than 3; and (5) able to understand and follow instructions related to the experiment. Exclusion criteria for the stroke subjects included: (1) a history of multiple strokes or bilateral involvement; (2) presence of contracture that would limit full elbow range of motion on the impaired side; (3) presence of cocontractions between flexors and extensors at rest during clinical assessment; (4) spatial-visual neglect; (5) elbow flexor MAS score of 3 or 4 in the impaired elbow that would make it difficult to position the elbow and forearm in a customized device; and (6) taking baclofen or any medication which could alter the severity of muscle spasticity. The detail information of the stroke subjects is listed in Table 1. The Committee for the Protection of Human Subjects at the University of Texas Health Science Center at Houston approved the procedures of this study. All participants provided written informed consent before participating in the study.

**Table 1:** Stroke subjects list.

**Subject**	**Age (yr)**	**Gender**	**History of stroke (Months)**	**Paretic side**	**Dominant side**	**Elbow flexor MAS**	**Wrist flexor MAS**	**Finger flexor MAS**	**Impaired biceps MVC (N-m)**	**Non-impaired biceps MVC (N-m)**	**Lesion type and site**
Stroke_1	61	F	100	R	R	1	2	3	8.6	31.2	Hemorrhgic, Left MCA
Stroke_2	64	F	185	R	R	1+	2	1	17.6	37.3	Ischemic
Stroke_3	70	M	66	R	R	1+	2	2	16.6	48.5	Ischemic, Left frontotemporal
Stroke_4	70	M	84	R	R	1+	2	1	9.3	37.43	Ischemic, Left caudate
Stroke_5	72	M	82	R	R	1	0	1	26	39	Ischemic, Left caudate
Stroke_6	59	F	100	L	R	1	0	1	16.4	26.9	Ischemic, Right MCA
Stroke_7	72	M	64	L	R	1+	2	3	6.24	23.85	Ischemic, Right MCA
Stroke_8	62	M	7	R	R	2	1	1	16	44.6	Hemorrhagic
Stroke_9	55	F	87	L	R	2	1	1	35.1	41.9	Ischemic, Left basal ganglia and thalamic
Stroke_10	59	M	82	L	R	1	1+	1+	7	39	Ischemic, Right MCA
Stroke_11	56	M	30	L	R	1	0	1	10	29	Hemorrhagic

*yr: year; F: female; M: male; R: right; L: left; MCA: middle cerebral artery*.

### Experimental tasks

In this study, we aimed to examine motor overflow and its relations to spasticity and weakness in chronic stroke. Motor overflow was assessed from the contracting biceps muscle (iBiceps) to the contralateral resting biceps (cBiceps), ipsilateral flexor digitorum superficialis (iFDS), and contralateral FDS (cFDS) muscles in healthy and stroke subjects. There were four motor overflow tasks as follows: (1) Dominant elbow flexion (_D_EF) tasks for healthy subjects; (2) Non-dominant elbow flexion (_ND_EF) tasks for healthy subjects; (3) Impaired elbow flexion (_IP_EF) tasks for stroke subjects; (4) Non-impaired elbow flexion (_NIP_EF) tasks for stroke subjects. In addition, spasticity and weakness of elbow flexors were quantified using an established biomechanical paradigm as described below.

Each subject was comfortably seated in an upright position on a height-adjustable chair. The subject was asked to keep a symmetric position with their both arms under the following configuration. The shoulder joint was kept approximately in 45° of flexion and 30° of abduction, while the elbow joint was kept in 90° flexion. The forearm was placed in neutral position. The active arm was placed in a customized arm device, while the forearm was secured against two adjustable metal plates with a padded strap approximately 2-4 inches proximal from the wrist. The resting arm was rested on a height-adjustable table. A 20-inch monitor (Model: 2001FP, Dell Computer Corp., Texas, USA) was used to provide visual feedback of the force produced by elbow flexion and the target force. The monitor was placed about 1 meter in front of the subject at eye level. All subjects reported that they could see the display clearly. Subjects performed the following tasks.

1) Muscle strength testing: Maximum voluntary contraction (MVC) force was estimated 3 times for elbow flexion and grip strength of both sides, respectively. The subjects were asked to produce a maximum force for 3–5 s. The highest force among 3 attempts was considered the MVC force. Maximum force of elbow flexion was used to pre-define the target force in the main experiments. One-minute rest was provided between consecutive MVC attempts.
2) Motor overflow tasks: Before a trial began, a target force level was provided as a red horizontal line in the middle of the monitor. The real-time force signal was provided as a white trace on the screen. It ran from left to right during each 12 s trial. For each trial, the subjects were asked to wait about 1 s (to show the baseline) and then increase their elbow isometric contraction force to reach the target within 2 s. Subjects were encouraged to match the white line (force) with the red line (target) as closely as possible throughout the trial. One to three practice trials were given to the subjects for familiarization of the force task.

**Figure 2:**
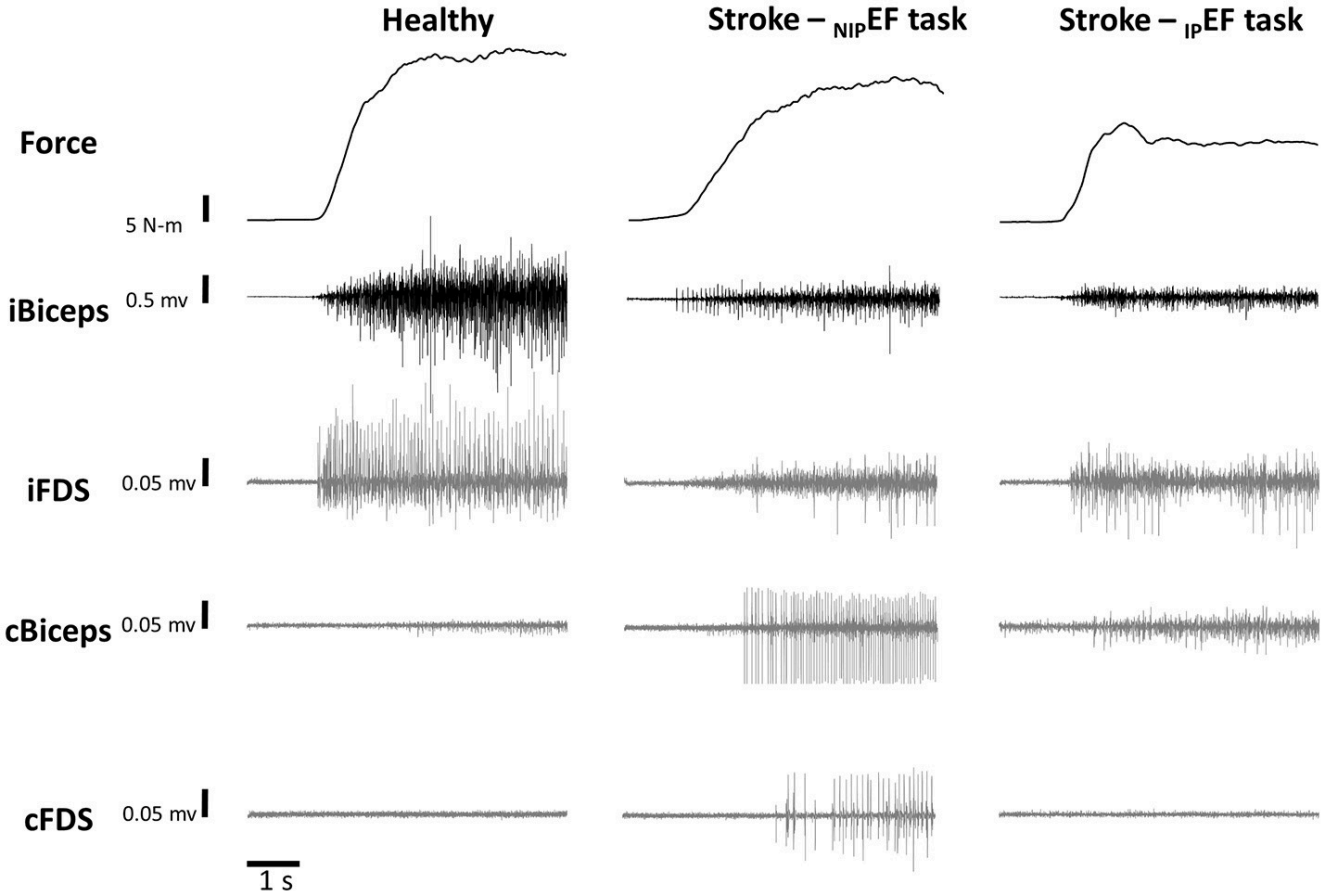
Representative trials of force and EMG during a 60% MVC _ND_EF task of one healthy subject and 60% MVC _NIP_EF and _IP_EF tasks of one stroke subject. Note that the scale for the iBiceps is 0.5 mV and the scales for the overflow muscles (iFDS, cBicep, and cFDS) are 0.05 mV.

Figure 2 illustrates the raw data of representative _D_EF task from one healthy subject, _NIP_EF task and _IP_EF task from one representative stroke subject. All the healthy subjects were asked to perform _D_EF and _ND_EF tasks with 10, 30, and 60% of the MVC forces as the targets, respectively. All the stroke subjects were asked to perform _IP_EF and _NIP_EF tasks with 10, 30, and 60% of the MVC forces as the targets, respectively. All subjects were asked to perform six trials for each force level. The order of the three force levels was randomized for each subject. During isometric contraction, subjects were explicitly instructed to keep other muscles relaxed. Adequate rest breaks were allowed between trials to minimize any possible fatigue effect.

3) Quantification of elbow flexor spasticity: Only the impaired elbow of stroke subjects was passively stretched in this task. An established experimental paradigm was used (25, 28). Subjects were asked to relax during the passive stretch tasks. The servo motor moved the forearm from elbow flexion 50° to full extension (0°) and then moved it back to the initial position, i.e., the range of the stretch was elbow flexion 50° to full extension. The servo motor was set motionless 2 s before the stretch, 2 s after reaching full extension, and 2 s after moving back to the initial position during a trial. The total length of a trial depended on the stretch speed. There were two different stretch speeds: 5 and 100°/s. Each task was performed three times in a roll, and the order of the two tasks was randomized between subjects. There was approximately 1 minute rest between trials.

### Data collection

Elbow flexion force was measured using a torque sensor (Model: TRS-500, Transducer Techniques, Temecula, CA, USA). The sensor was located in line with the center of the rotation of the active elbow joint. During MVC task, Grip strength was measured using a hand dynamometer (Jamar Plus+; Sammons Preston, Rolyon, Bolingbrook, IL). Surface EMG electrodes (Delsys 2.1 Single Differential Configuration, Delsys Inc., Boston, MA, USA) were placed on the biceps and FDS muscles bilaterally according to the European Recommendations for Surface Electromyography (29). The EMG signals were collected through a Bagnoli EMG system (Delsys Inc.), amplified 1000 times. All the collected signals were sampled at 1,000 Hz with a NI-DAQ card (Model: PCI-6229, National Instruments, Austin, TX, USA) and stored on a personal computer.

### Data analysis

Data was analyzed off-line using custom-written Matlab programs (MathWorksTM Inc., Natick, Massachusetts, USA). The raw torque signal was low-pass filtered at 10 Hz with a fourth-order, zero-lag Butterworth digital filter before further analysis. The following parameters were calculated:

#### Root-mean-square (RMS) EMG and motor overflow

Before RMS EMG calculation, the raw EMG signal was detrended in order to remove the offset before further analysis. For MVC trials, RMS EMG was calculated over a 2-s window centered on the peak force. For submaximal isometric tasks, a 2-s segment of EMG signals in the middle of each trial was used to standardize the analysis as used in our recent studies (15, 30, 31). Subjects were able to generate a steady force output during this time window. RMS EMG values were calculated for biceps and FDS on both sides for each trial. RMS EMG values were further normalized to the MVC trials of the corresponding muscles (nEMG).

As shown in representative trials in Figure 2, there were EMG activities in resting non-contracting muscles (ipsilateral FDS, contralateral biceps, contralateral FDS) during unilateral isometric elbow flexion tasks in both healthy and stroke subjects. Motor overflow was defined as nEMG in these muscles in this study, reflecting involuntary activation of the resting muscles.

#### Reflex torque

A resistance torque was generated during the passive rotation of the elbow joint by a servomotor. The total resistance torque of a passive stretching task was calculated as the differences between the mean torque over a 200 ms window prior to stretching (the baseline) and the highest torque during the stretch as described in our recent study (28). The total resistance torque includes both reflex and non-reflex resistances. The total resistance torque to slow stretching (5°/s) is considered to reflect the passive and non-reflex property of spastic muscles. The difference in the total resistance torque between fast (100°/s) and slow stretching thus represents the reflex component of spastic muscles (24, 28, 32). Therefore, the reflex torque was calculated as the difference in the total resistance torque between 5 and 100°/s stretch speeds.

#### Weakness

Peak values of individual MVC tasks were measured on both sides for elbow flexion and grip tasks. The weakness of a task on the impaired side was quantified as the percent of the MVC force of the impaired muscle with reference to the MVC force on the contralateral side.

#### EMG-EMG coherence

Before EMG-EMG coherence calculation, the raw EMG signal was detrended in order to remove the offset. Coherence was calculated between iBiceps muscle with the other three muscles, respectively. For all healthy subjects, we pooled all the EMG signals from _D_EF and _ND_EF tasks for iBiceps, cBiceps, iFDS, and cFDS muscles, respectively. For all stroke patients, we pooled the EMG signals for iBiceps, cBiceps, iFDS and cFDS muscles for _IP_EF and _NIP_EF tasks, separately. The EMG power spectrum of each muscle was calculated with a 500 ms (500 data points) epoch, with zero overlap moving windows using the built-in fft function in Matlab. The same 2-s window used for RMS EMG calculation was used to calculate the coherence for each trial, so the total epochs used in EMG-EMG coherence calculation for healthy subjects were 1,584 (4 epochs/trial × 6 trials × 3 force levels × 2 sides of upper limbs × 11 subjects). For _IP_EF task in stroke subjects, there were total 792 epochs (4 epochs/trial × 6 trials × 3 force levels × 11 subjects) between iBiceps and cBiceps and between iBiceps and cFDS muscles, while there were only 648 (4 epochs/trial × 6 trials × 3 force levels × 9 subjects) epochs between iBiceps and iFDS muscles due to no voluntary contraction of impaired FDS muscle in two stroke subjects. For _NIP_EF task in stroke patients, similar to _IP_EF task, there were total 792 epochs (4 epochs/trial × 6 trials × 3 force levels × 11 subjects) between iBiceps and cBiceps and between iBiceps and iFDS muscles, while there were only 648 epochs (4 epochs/trial × 6 trials × 3 force levels × 9 subjects) between iBiceps and cFDS muscle due to no voluntary contraction of impaired FDS muscle in two stroke patients. We used the following equation to calculate EMG-EMG coherence:

$$\begin{array}{l} C_{xy}\left(f\right)=\;\frac{{\left|P_{xy}\left(f\right)\right|}^{2}}{P_{xx}\left(f\right)P_{yy}\left(f\right)} \end{array}$$

Where C_xy_ represents the coherence between EMG signal *x* and *y*, and *f* is the frequency. *P*_*xx*_ and *P*_*yy*_ represent autospectra for signal *x* and *y*, while *P*_*xy*_ represents the cross spectrum of signal x and y. The EMG-EMG coherence calculation described above is one of the standard methods in the literature (17, 33, 34). Coherence values were calculated between 0 and 350 Hz. We focused on significant coherence during 6–12 Hz (alpha band), 13–30 Hz (beta band), and 30–60 Hz (gamma band) frequency bands because they have been previously associated with cortical or subcortical origins (17–20).

### Statistical analysis

The dependent variables in this study were: (1) MVC force; (2) normalized RMS EMG (nEMG); (3) reflex torque; (4) weakness; and (5) EMG-EMG coherence. Paired *t*-test (two-tailed) was used to test the difference between dominant and non-dominant MVC forces in healthy subjects, and impaired and non-impaired MVC forces in stroke subjects, respectively. Two-way repeated measure ANOVAs were used to compare the effect of dominance for the nEMG parameters in healthy subjects with factors of SIDE (dominant or non-dominant) and FORCE LEVEL (10, 30, and 60% of MVC). Data were then averaged from two sides for healthy subjects for further comparisons with stroke data since no statistical significance was found. Two-way mixed ANOVAs were used to compare the differences between healthy and stroke subjects for the nEMG parameters with a between-group factor of GROUP (healthy and stroke) and a within-group factor of FORCE LEVEL (10, 30, and 60% of MVC) for each side (impaired, _IP_GROUP and non-impaired, _NIP_GROUP) separately.

In order to test the correlation between severity of motor impairment and motor overflow, Pearson coefficient correlations (*r*) were calculated between severity parameters (reflex torque and weakness) and nEMG of cBicep, iFDS, and cFDS muscles. For EMG-EMG coherence, because the different number of epochs among different coherence calculations, the coherence value was considered significant when it was above two standard deviation of the mean coherence value between 0 and 350Hz during each pair of EMG-EMG coherence calculation (33–35). All statistical analyses, except EMG-EMG coherence, were performed with the Statistica 13 software (StatSoft Inc. CA, USA). The alpha level for all statistical tests was 0.05. Data are reported as mean ±*SD* within the text and as mean ± SEM in the figures. Only the significant main effects are presented, unless otherwise noted.

## Results

### MVC tasks

There was no significant difference in MVC values between the dominant and non-dominant sides of the healthy subjects for elbow flexion tasks (42.1 ± 10.7 N-m vs. 37.9 ± 6.9 N-m; *p* = 0.14). However, healthy subjects exhibited smaller grip strength with the non-dominant hand (36.0 ± 9.2 Kg) compared to the dominant hand (43.8 ± 9.7 Kg; *p* < 0.01). Moreover, stroke subjects exhibited significant lower elbow flexion (IP: 15.3 ± 8.8 N-m; NIP: 36.2 ± 7.7 N-m, *p* < 0.01) and grip (IP: 12.2 ± 10.2 Kg; NIP: 33.7 ± 7.3 Kg, *p* < 0.01) strength on the impaired (IP) side compared to the non-impaired (NIP) side. There were two stroke subjects who cannot perform voluntary grasping, so the averaged grip strength of the impaired side was from 9 stroke subjects.

### Motor overflow in healthy subjects

**Figure 3:**
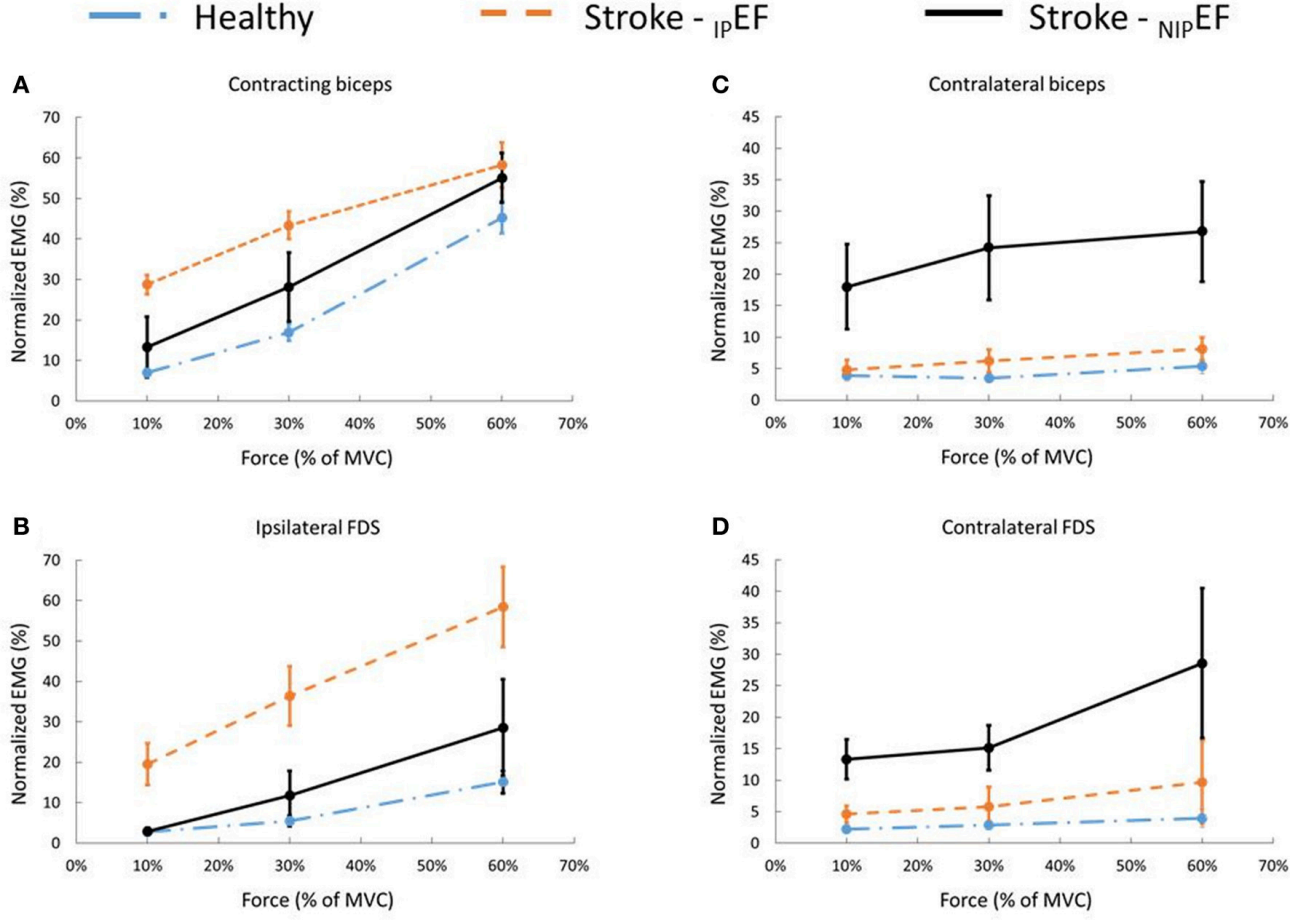
Muscle activity during _D_EF and _ND_EF tasks for healthy subjects and _IP_EF and _NIP_EF tasks for stroke subjects during 10, 30, and 60% of the MVC contraction task. **(A)** iBiceps muscle (contracting muscle) activity of stroke patients during _IP_EF and _NIP_EF tasks and the averaged iBiceps muscle activity of healthy subjects. **(B)** iFDS muscle activity of stroke patients during _IP_EF and _NIP_EF tasks and the averaged iFDS muscle activity of healthy subjects. **(C)** cBiceps muscle activity of stroke patients during _IP_EF and _NIP_EF tasks and the averaged cBiceps muscle activity of healthy subjects. **(D)** cFDS muscle activity of stroke patients during _IP_EF and _NIP_EF tasks and the averaged cFDS muscle activity of healthy subjects.

EMG activities of the contracting biceps (iBiceps) of each side increased with the level of force. A two-way repeated measures ANOVA showed that there was a main effect of FORECE LEVEL on the nEMG of iBiceps [*F*_(2, 20)_ = 125.9, *p* < 0.01] (Figure 3A). The nEMG increased with force levels of the tasks for iBiceps on each side (pooled data: 10% MVC: 7.0 ± 4.6%; 30% MVC: 16.9 ± 9.0%; 60% MVC: 45.3 ±17.0%). However, there were no significant main effects of SIDE or SIDE x FORCE LEVEL interactions.

Motor overflow to the non-contracting, resting muscles, or the nEMG, showed a similar pattern for cBiceps and iFDS (Figures 3B,C). There was a significant main effect of FORCELEVEL on the nEMG of cBiceps [*F*_(2, 20)_ = 5.3, *p* = 0.01], and iFDS [*F*_(2, 20)_ = 26.0, *p* < 0.01]. Motor overflow increased with the level of isometric elbow flexion of iBiceps, respectively for cBiceps (10% MVC: 3.5 ± 2.9%; 30% MVC: 3.5 ± 2.2%; 60% MVC: 5.4 ± 4.9%), and iFDS (10% MVC: 2.7 ± 3.2%; 30% MVC: 5.5 ± 5.8%; 60% MVC: 15.1 ± 11.4%). However, motor overflow to the resting cFDS was not level-dependent. The nEMG of cFDS was 2.3 ± 3.4% at 10% MVC; 2.9 ± 3.1% at 30% MVC; 3.9 ± 5.8% at 60% MVC (*p* = 0.19), respectively. Furthermore, there were no significant effects of SIDE or SIDE x FORCE LEVEL interactions on the nEMG of cBicep, iFDS, and cFDS (all *p* > 0.1). These results showed that muscle activity and motor overflow pattern was similar between _D_EF and _ND_EF tasks in healthy subjects. The nEMG values were averaged between _D_EF and _ND_EF tasks for each muscle under each force levels. The averaged nEMG values were used to compare with stroke subjects for the rest of the analyses.

### Motor overflow in stroke subjects

There was a similar pattern of force level-dependent increase in normalized EMG activities (nEMG) in both impaired and non-impaired side of stroke subjects (Figure 3A). There was no significant difference in nEMG of the contracting biceps between the non-impaired side of stroke subjects and healthy controls (all *p* > 0.1). However, the nEMG of the contracting biceps was greater in the impaired side of stroke subjects than in healthy subjects. There were main effects of _IP_GROUP [*F*_(1, 20)_ = 7.8, *p* = 0.01], FORCELEVEL [*F*_(2, 40)_ = 96.1, *p* < 0.01], and _IP_GROUP x FORCELEVEL interactions [*F*_(2, 40)_ = 3.8, *p* = 0.03] (Figure 2A). *Post-hoc* analyses indicated that both healthy and stroke subjects exhibited higher nEMG with the increase of generated force (Healthy, pooled data 10% MVC: 7.0 ± 3.5%; 30% MVC: 16.9 ± 6.7%; 60% MVC: 45.3 ± 13.0%; Stroke: 10% MVC: 28.7 ± 24.6%; 30% MVC: 43.3 ± 28.1%; 60% MVC: 58.2 ± 20.2% on the impaired side). Furthermore, stroke subjects exhibited significantly higher iBiceps nEMG during 10% and 30 % MVC tasks on the impaired side compared with healthy subjects.

Motor overflow was observed in the resting muscles during both _IP_EF and _NIP_EF tasks. As shown in representative trials, the pattern of motor overflow was different, depending on the task, i.e., which arm is the active arm (Figure 2). However, _IP_EF and _NIP_EF tasks generated different patterns of motor overflow to the resting muscles in ipsilateral and contralateral sides (Figures 3B–D). Note that there were two stroke subjects who cannot perform voluntary grip force, so there were only 9 subjects who had nEMG for iFDS during _IP_EF tasks and cFDS during _NIP_EF tasks.

When the impaired biceps was contracting during the _IP_EF tasks, there was significantly greater motor overflow to the distal finger flexors (iFDS), but similar motor overflow to the contralateral resting muscles (cBiceps and cFDS) as compared to healthy controls (Figure 3). Two-way ANOVA tests showed significant effects of _IP_GROUP [***F***_(1, 18)_ = 18.7, *p* < 0.01], FORCELEVEL [*F*_(2, 36_ = 31.4, *p* < 0.01], and _IP_GROUP × Force interaction [***F***_(2, 36)_ = 8.3, *p* < 0.01] for iFDS nEMG (Figure 3B). *Post-hoc* analyses indicated that stroke subjects exhibited higher iFDS nEMG during 10% (19.5 ± 17.2% vs. 2.7 ± 2.5%), 30% (36.4 ± 24.3% vs. 5.5 ± 4.4%), and 60% (58.4 ± 33.2% vs.15.1 ± 9.2%) MVC _IP_EF tasks compared with healthy subjects. Furthermore, although the iFDS nEMG increased with the force level for both stroke [*F*_(2, 16)_ = 15.9, *p* < 0.01] and healthy [*F*_(2, 20)_ = 26.0, *p* < 0.01] subjects, the significant interaction indicated that the increment slope was sharper in stroke subjects compared with healthy subjects. There were no significant differences between stroke and healthy subjects in terms of cBiceps and cFDS nEMG (all *p* > 0.1).

When the non-impaired biceps was contracting during the _NIP_EF tasks, there was significantly greater motor overflow to the contralateral spastic muscles (cBiceps and cFDS) of the impaired side, but similar motor overflow to the distal finger flexors (iFDS) as compared to healthy subjects. There were significant effects of _NIP_GROUP [*F*_(1, 20)_ = 6.2, *p* = 0.02), FORCE LEVEL [*F*_(2, 40)_ = 8.0, *p* < 0.01], and _NIP_GROUP x FORCE LEVEL interactions [*F*_(2, 40)_ = 4.0, *p* = 0.03] for cBiceps nEMG (Figure 3C). *Post-hoc* analyses revealed that stroke subjects exhibited higher cBicep nEMG during 60% MVC _NIP_EF task compared with healthy subjects (Stroke: 26.8 ± 26.2%; Healthy: 5.4 ± 3.9%). Similarly, there were main effects of _NIP_GROUP [*F*_(1, 18)_ = 8.2, *p* < 0.01), FORCE LEVEL [*F*_(2, 36)_ = 6.7, *p* < 0.01], and _NIP_GROUP x FORCE LEVEL interactions [*F*_(2, 40)_ = 4.0, *p* = 0.03) for cFDS nEMG (Figure 3D). *Post-hoc* analyses indicated that stroke subjects exhibited higher cFDS nEMG during 10% (19.5 ± 17.2% vs. 2.7 ± 2.5%), 30% (36.4 ± 24.3% vs. 5.5 ± 4.4%), and 60% (58.4 ± 33.2% vs. 15.1 ± 9.2%) MVC _NIP_EF tasks as compared with healthy subjects. Furthermore, the cFDS nEMG increased with the force level in stroke subjects [*F*_(2, 16)_ = 9.3, *p* < 0.01], but not healthy subjects [*F*_(2, 20)_ = 1.8, *p* = 0.18].

### Correlation between impairment and motor overflow

Linear correlation analyses between impairment severity parameters (reflex torque and weakness) and motor overflow parameters (nEMG of cBicep, iFDS, and cFDS muscles) were performed to investigate whether the impairment level correlated with altered motor overflow. The correlation coefficients were summarized in Table 2. When the biceps on the non-impaired side was contracting during _NIP_EF tasks, correlation coefficient between motor overflow and spasticity of the biceps (reflex torque) was moderate to high. The correlation was very high across all force levels for cFDS. There was no consistent pattern of correlation between motor overflow and reflex torque during _IP_EF tasks. No consistent correlation between motor overflow and weakness was observed. Note that we excluded two stroke subjects for the correlation analyses. One of the stroke subjects cannot relax during the passive stretch tasks, and the 5°/s stretch data was missing in another subjects, so there were only 9 subjects available for the calculation of correlation between reflex torque and motor overflow. Furthermore, due to no voluntary contraction of impaired FDS muscles as we mentioned above, we also excluded those two subjects from correlation analyses for cFDS during _NIP_EF tasks and iFDS during _IP_EF tasks (*n* = 7).

**Table 2:** Pearson correlation coefficients of reflex torque and weakness with nEMG of cBiceps, iFDS, and cFDS.

		**NIP contracting**			**IP contracting**		
		**cBiceps**	**iFDS**	**cFDS**	**cBiceps**	**iFDS**	**cFDS**
Reflex Torque	10% MVC	0.44 *n* = 9	0.47 *n* = 9	0.87^*^n = 7	−0.32 *n* = 9	0.49 *n* = 7	−0.21 *n* = 9
	30% MVC	0.43 *n* = 9	0.47 *n* = 9	0.79^*^n = 7	0.37 *n* = 9	−0.39 *n* = 7	0.43 *n* = 9
	60% MVC	0.49 *n* = 9	0.51 *n* = 9	0.84^*^n = 7	−0.31 *n* = 9	0.45 *n* = 7	0.47 *n* = 9
Weakness	10% MVC	0.32 *n* = 11	−0.12 *n* = 11	−0.31 *n* = 9	−0.11 *n* = 11	−0.17 *n* = 9	−0.12 *n* = 11
	30% MVC	0.33 *n* = 11	−0.15 *n* = 11	−0.33 *n* = 9	−0.11 *n* = 11	−0.14 *n* = 9	−0.09 *n* = 11
	60% MVC	−0.31 *n* = 110	−0.04 *n* = 11	0.52 *n* = 9	0.33 *n* = 11	0.42 *n* = 9	−0.08 *n* = 11

* *Indicates statistical significant correlation (p < 0.05)*.

### Coherence between contracting biceps and resting muscles

**Figure 4:**
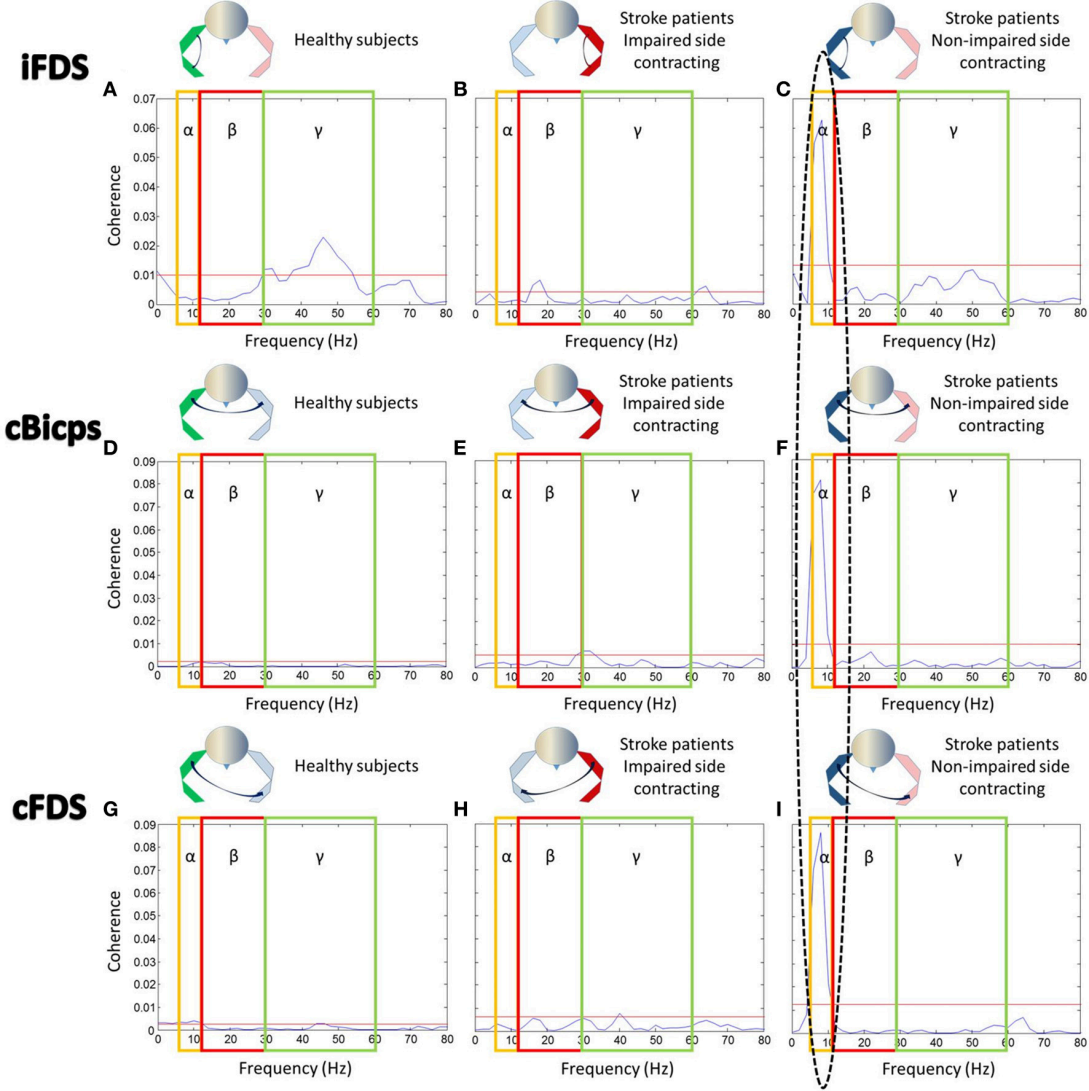
EMG-EMG coherence between contracting biceps and other resting muscles. Note that the ellipse highlights the significant coherence in the alpha band. Yellow boxes, red boxes, and blue boxes indicated the range of alpha band, beta band, and gamma band, respectively. Red lines indicated significant level for each figure. Coherence between **(A)** iBiceps and iFDS muscles in healthy subjects, (**B**) iBiceps and iFDS muscles during _IP_EF tasks in stroke patients, **(C)** iBiceps and iFDS muscles during _NIP_EF tasks in stroke patients, **(D)** iBiceps and cBiceps muscles in healthy subjects, **(E)** iBiceps and cBiceps muscles during _IP_EF tasks in stroke patients, **(F)** iBiceps and cBiceps muscles during _NIP_EF tasks in stroke patients, **(G)** iBiceps and cFDS muscles in healthy subjects, **(H)** iBiceps and cFDS muscles during _IP_EF tasks in stroke patients, and **(I)** iBiceps and cFDS muscles during _NIP_EF tasks in stroke patients.

*Coherence between contracting biceps and ipsilateral FDS (Figures* *4A–C**, upper row)*: For healthy subjects, there was significant coherence in the gamma band between 30 and 54Hz (coherence power range 0.011–0.023) (Figure 4A). During _IP_EF tasks in stroke subjects, there was significant coherence in the beta band between 18 and 20Hz (range: 0.006–0.008) (Figure 4B). During _NIP_EF tasks in stroke patients, the significant coherence shifted further to the alpha band at 6–10Hz (range: 0.015–0.063), although coherence level was relatively high in the gamma band as well (Figure 4C).

*Coherence between contracting biceps and contralateral biceps (Figures 4D–F, middle row)*: For healthy subjects, there was no significant coherence in all three frequency bands (Figure 4D). During _IP_EF tasks in stroke patients, there was significant coherence in the beta band and gamma bands between 28 and 32Hz (range: 0.005–0.007) (Figure 4E). During _NIP_EFs tasks in stroke patients, the significant coherence happened in the alpha band between 6 and 10Hz (range: 0.015–0.081) (Figure 4F).

*Coherence between contracting biceps and contralateral FDS (Figures 4G–I, lower row)*: for healthy subjects, there was significant coherence in the alpha and gamma bands between 6 and 12Hz (range: 0.003–0.004) and 44–60Hz (0.003), respectively (Figure 4G). During _IP_EF tasks in stroke subjects, there was significant coherence in the gamma band at 40 Hz (0.008) (Figure 4H). During _NIP_EF tasks in stroke subjects, the significant coherence happened in the alpha band between 6 and 10Hz (range: 0.02–0.09) (Figure 4I).

## Discussion

**Figure 5:**
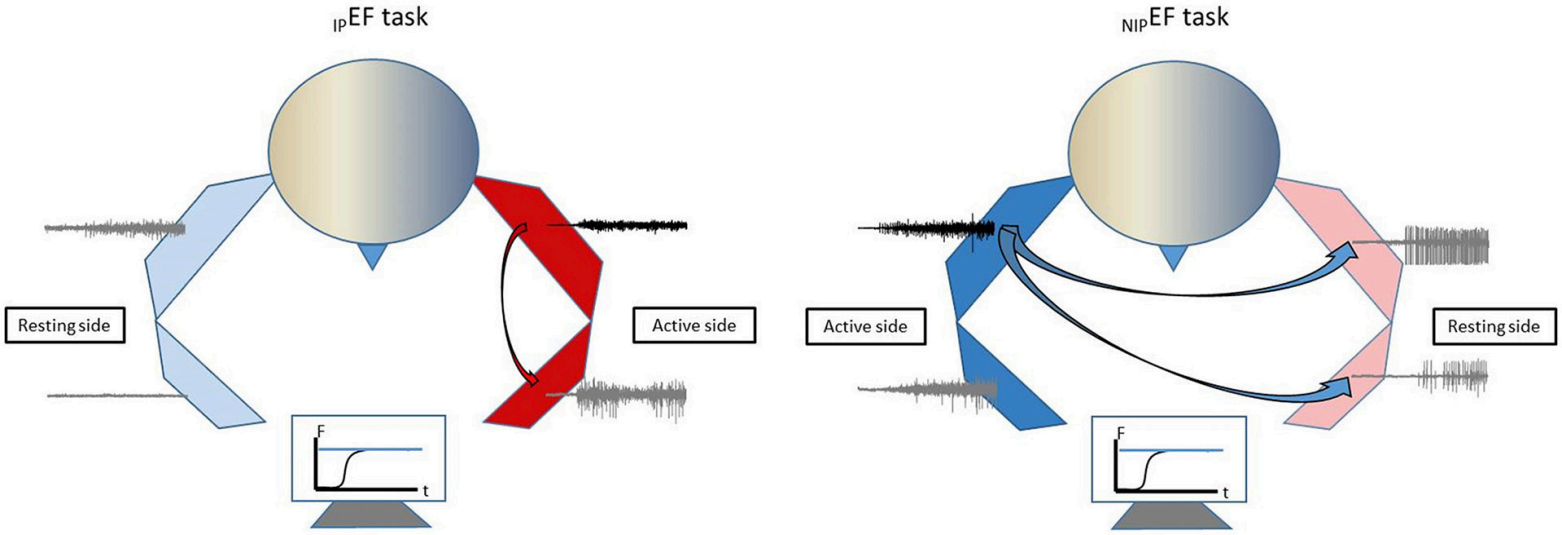
Schematic illustration of motor overflow patterns in chronic stroke.

In this study, stroke survivors with spastic elbow and finger flexors and healthy controls performed unilateral isometric elbow flexion tasks at 10, 30, and 60% of MVC. Motor overflow was quantified as EMG activity of a resting muscle normalized to its corresponding MVC value (nEMG). We found that stroke subjects exhibited greater motor overflow to the impaired side in general, either from the proximal to distal muscle during elbow flexion on the impaired side or from the non-impaired to impaired side during elbow flexion on the non-impaired side. This pattern of motor overflow is schematically presented in Figure 5. Exaggerated motor overflow increased as the level of voluntary contraction increased. Correlation between exaggerated motor overflow and severity of spasticity (reflex torque) was consistently at moderate to high levels. No consistent correlation between motor overflow and weakness was observed. Furthermore, exaggerated motor overflow has a significantly high EMG-EMG coherence in the alpha band with the contracting biceps muscles on the non-impaired side. No qualitative difference in EMG-EMG coherence was observed between healthy subjects and stroke subjects during unilateral elbow flexion tasks on the impaired side. Findings from comprehensive analyses of motor overflow (nEMG, correlation with spasticity and weakness, and EMG-EMG coherence) shed light on the mechanisms of this phenomenon.

### Correlation between motor overflow and post-stroke spasticity

In the present study, our findings of greater and force level-dependent motor overflow in stroke subjects are consistent with our previous studies (15, 16) and other reports in the literature (3, 8). Our results also showed that stroke subjects who had greater reflex torque exhibited more motor overflow using an established biomechanical approach, i.e., there was moderate to high correlation between motor overflow and spasticity. This finding is consistent with many previous studies (2, 3, 6). However, two studies did not report any significant correlation between motor overflow and spasticity (1, 4). In Ada and O'Dwyer paper (1), stroke subjects with either spasticity or contracture were recruited. Associated reaction (i.e., motor overflow) was determined by the presence of muscle activity and was quantified as elbow flexor torque produce on the impaired side during moderate elbow flexion tasks on the contralateral side. Spasticity was identified by the presence of abnormal reflex activity. They reported presence of contracture in half of the stroke subjects. The incidence of motor overflow was the same as of spasticity. However, the correlation between spasticity and motor overflow was determined by non-parametric analysis. The relation between spasticity and motor overflow is likely affected by non-selective subject enrollment and non-quantitative assessment of spasticity. In another study (4), Bhakta et al. found that the abnormal motor overflow of peak grip force on the impaired side during maximum voluntary grip on the contralateral side was not correlated with the summed MAS over elbow, wrist and finger flexors on the impaired side. The use of summed MAS was not justified and may have changed the relations.

### Possible mechanisms of motor overflow and spasticity—a common pathophysiological process

A significant coherence in the alpha band between EMGs of the contracting biceps muscle on the non-impaired side and other EMGs of resting muscles (ipsilateral distal FDS, contralateral spastic biceps muscles, and FDS) suggests a subcortical origin of motor overflow, likely from the reticulospinal drive (21). Alternatively, the alpha band coherence could also be originated from spinal mechanisms (36). In this study, Monkeys were trained to perform slow unilateral finger movements. Coherence between local field potentials and movement acceleration was found to be significant at 6–13 Hz for all tested areas in cerebellar nuclei, pontomedullary reticular formation, and the spinal cord. Further detailed analysis revealed that convergence of antiphase oscillations of cortical and subcortical descending inputs at the spinal cord motoneuronal level limited motor drive to muscles at a frequency at 10 Hz. This phase cancelation mechanism at the spinal cord was viewed to improve movement precision. However, this mechanism is not likely to explain involuntary EMG activity of the resting muscle on the contralateral side.

A different phenomenon was observed during bimanual tasks (37). Cortical activity using EEG and muscle activity of bilateral flexor pollicis brevis using surface EMG were recorded when healthy adult subjects performed bimanual force coordination tasks. Corticomuscular (EEG and EMG) coherence was significant in the beta band (16–30 Hz), consistent with the corticospinal beta-band drive for the contralateral limb muscles. Intermuscular coherence between bilateral EMGs was only observed in the alpha band (5-12Hz) and increased with bimanual coordination. The alpha band coherence was subcortical in origin. These results are supportive that multiple parallel pathways are involved in motor tasks (38). In case of stroke that damages primary motor cortex and/or its descending corticospinal pathways, descending projections of subcortical origins, particularly medial reticulospinal projections, are unopposed and upregulated for possible compensation (39, 40). As such, the alpha-band coherence in this study is most likely a release from cortical control, and is consistent with a reticulospinal drive (21). This view is supported by the findings from a recent longitudinal study (41).

It is known that there is bilateral activation of reticulospinal pathways (42–49). Motor overflow could be attributed to the accompanied non-selective activation of reticulospinal projections during voluntary movement. In particular, this non-selective activation is likely to bring spinal motor neurons which are commonly hyperexcitable but at the sub-threshold levels to fire for these spastic muscles, or to increase spontaneous firing activities of these neurons (50, 51). In this study, we observed motor overflow from proximal to distal muscles on the impaired side, and to both proximal and distal muscles on the impaired side during voluntary flexion on the non-impaired side. Reticulospinal hyperexcitability is considered a possible underlying pathophysiology of post-stroke spasticity (26, 27). Our results of high correlation between motor overflow and spasticity further support the shared underlying mechanism of these two phenomena.

### Rehabilitation relevance

Better understanding of mechanism of motor overflow and its relations with spasticity helps guide management of these clinical problems. Reticulospinal hyperexcitability is a maladaptive plastic change in the course of post-stroke motor recovery. In our previous study, motor overflow was only in the patients who have spastic hemiplegia (spastic stage), but not in those with good motor recovery and no spasticity (recovered stage), suggesting the important role of reticulospinal pathway in the development of spasticity, rather than contributions to motor recovery after stroke (16). Even in the spastic stages where stroke survivors have various degrees of spasticity, reticulospinal hyperexcitability does not contribute to development of muscle strength. Recent studies have demonstrated involuntary activation of spastic muscles during and after voluntary contraction (30, 50, 51). When reticulospinal pathways are stimulated by acoustic stimulation during sustained elbow flexion, the induced force increase is similar between stroke survivors with spasticity as compared to healthy subjects (52).

Motor overflow needs to be suppressed if the problem is exaggerated and intervenes activities of daily living. As shown in Figure 1, this patient has suffered from exaggerated motor overflow to his spastic right arm and hand. Every time when he exerts moderate to strenuous effort with his left hand, such as lifting a heavy object, working out in the gym, his right arm and wrist and fingers flexes synergistically. His right wrist reaches to his chest at times. Apparently, this exaggerated motor overflow further affects posture, and walking. Interventions, such as Botulinum toxin injection to his left elbow, wrist and finger flexors, help reduce motor overflow, and improve comfort in performing activity of daily living.

It is important to pay attention of the role of motor overflow in bilateral tasks. Motor overflow may present as bilateral synergistic coupling (53, 54). In our previous study (54), when hemiparetic stroke survivors are instructed to flex bilateral elbow joints simultaneously to match a visually guided constant force (i.e., target), the paretic side and the contralateral side are able to generate and share the total force proportionally with reference to individual maximum strength. When the visual gain of force from one side is altered, up to 8 times easier (× 8) or harder (× 1/8), the force on the other side is able to adjust its force proportionally to match the visual target. In other words, the relation between forces of each side is maintained despite of alteration of visual gain for force from only one limb. Therefore, motor rehabilitation programs involving bilateral training need to be utilized with cautions in spastic hemiplegia.

There are limitations of this study. No age-matched controls were tested, though commonly recommended. Generally speaking, the patterns from the young, healthy group data are used as a reference point. We expect that these patterns would be the same if age- and gender matched controls were enrolled in the study, since pathological patterns of motor overflow should not be expected in neurologically intact healthy controls as we discussed in the Introduction section. In this study, the patterns of EMG-EMG coherence and motor overflow were similar between the non-impaired side of stroke subjects and the healthy controls. Such findings support our expectations. Due to required isometric elbow flexion tasks on the impaired side, stroke subjects with more severe spasticity and motor impairment were not enrolled. The established biomechanical paradigm allowed quantitative grading and assessment of spasticity and correlation analysis between spasticity and motor overflow. Spastic co-contraction often occurs during elbow flexion on the impaired side. It is a missed opportunity to assess triceps activity during elbow flexion tasks. We understand that our study only provides indirect evidence regarding brainstem mechanisms for post-stroke motor overflow. However, localization of brainstem nuclei and pathways is not available even with most advanced neuroimaging techniques. Due to technical limitations, our results significantly advance our understanding of underlying mechanisms of this common phenomenon of post-stroke motor overflow.

## Conclusions

To summarize, our results demonstrated that unilateral elbow flexion caused diffuse motor overflow from proximal to distal muscles on the impaired side (within-limb motor overflow) and from non-impaired side to the impaired side (between-limb motor overflow) in stroke subjects There were moderate to high correlations between the severity of spasticity and motor overflow. Furthermore, both within-limb and between-limb EMG-EMG coherence analysis showed significant coherence in the alpha band, suggestive of subcortical origins. Collectively, these results suggest that diffuse motor overflow and spasticity share a common pathophysiological process. Reticulospinal hyperexcitability is likely the candidate to mediate these clinical phenomena.

## Author contributions

Y-TC, SGL, EM, PZ, and SL: Experimental design; Y-T C, SGL, SL: Data Collection; Y-T C, SGL, EM, PZ, and SL: Data Analysis and interpretation; Y-T C and SL: Manuscript Draft; Y-T C, SGL, EM, PZ, and SL: Discussion, intellectual inputs and final approval of the manuscript.

### Conflict of interest statement

The authors declare that the research was conducted in the absence of any commercial or financial relationships that could be construed as a potential conflict of interest.

## Abbreviations

cBiceps: contralateral biceps muscle
cFDS: contralateral flexor digitorum superficialis
_D_EF: dominant elbow flexion
FDS: flexor digitorum superficialis
iBiceps: contracting biceps muscle
iFDS: ipsilateral flexor digitorum superficialis
_IP_EF: impaired elbow flexion
_IP_GROUP: impaired group
MVC: maximum voluntary contraction
_ND_EF: non-dominant elbow flexion
nEMG: normalized electromyography
_NIP_EF: non-impaired elbow flexion
_NIP_GROUP: non-impaired group
RMS: root-mean-square
